# Melatonin receptor 1A gene polymorphism rs13140012 and serum melatonin in atherosclerotic versus non-atherosclerotic Egyptian ESRD patients: pilot study

**DOI:** 10.1016/j.heliyon.2020.e04394

**Published:** 2020-07-11

**Authors:** Aliaa Aly El Aghoury, Eman Tayae Elsayed, Noha Mohamed El Kholy, Mohamed Hesham El Nashar, Tarek M. Salem

**Affiliations:** a Department of Internal Medicine, Endocrinology Unit, Faculty of Medicine, University of Alexandria, Egypt; b Department of Clinical and Chemical Pathology, Faculty of Medicine, University of Alexandria, Egypt; c Department of Internal Medicine, Nephrology Unit, Faculty of Medicine, University of Alexandria, Egypt; d Department of Nephrology, Gamal Abd El Nasser Hospital, General Authority for Health Insurance, Alexandria Branch, Egypt

**Keywords:** Health sciences, Renal system, Internal medicine, Endocrinology, Clinical research, Diagnostics, Melatonin, Single nucleotide polymorphism (SNP), rs13140012, Atherosclerosis, End-stage renal disease (ESRD), Hemodialysis (HD), Carotid intima-media thickness (CIMT)

## Abstract

**Aim:**

To study the relationship between melatonin levels and Melatonin membrane receptor 1A (*MTNR1A*) SNP (rs13140012) in end-stage renal disease patients (ESRD) in Alexandria, Egypt on maintenance hemodialysis with or without atherosclerosis.

**Materials and methods:**

40 end-stage renal disease patients on regular hemodialysis were divided into 2 subgroups, one with (n = 20) and one without atherosclerosis (n = 20) and normal subjects (n = 40). Serum melatonin, carotid intimal medial thickness (CIMT) were measured. Melatonin membrane receptor 1A (*MTNR1A*) SNP (rs13140012) genotyping was done using 5'nuclease Allelic discrimination.

**Results:**

Serum melatonin was significantly lower in ESRD patients [1.6 to 11.30 (pg/mL) with a median of 2.5] than the control group [20.50 to 56.40 (pg/mL) with a median of 35.20]. Serum melatonin was significantly lower in atherosclerotic patients subgroup [1.6–2.50 (pg/mL) with a median value of 2.30] than non-atherosclerotic patients subgroup [2.0–11.30 (pg/mL) with a median of 4.9]. No significant association was found between serum melatonin and (*MTNR1A*) SNP (rs13140012) (p = 0.633).

**Conclusion:**

These results lead us to suggest that melatonin production is impaired in ESRD patients (included in this pilot study), and this impairment is more evident in atherosclerotic ESRD patients.

## 1 Introduction

Melatonin has a promising role as a cytoprotective molecule and having chronobiotic properties [1]. The pineal gland is its primary producer in circulation. Decreasing plasma melatonin is considered a hallmark of advancing age in humans [2].

Melatonin has antioxidant and scavenging effects independent of its receptors [3]. Melatonin exceeds that capacity of vitamin C and E to protect from oxidative damage [4]. Immunomodulation by melatonin includes pro-inflammatory and anti-inflammatory effects [5].

Melatonin receptors are G protein coupler receptors and are expressed in different parts of the CNS besides peripheral organs, including blood vessels, mammary glands, GIT, liver, kidney, and the immune system [6, 7].

The chronic state of kidney disease can be defined as an abnormality in either structure or function that has persisted for over three months, with an impact on the patient's general condition [8].

Progression from CKD to ESRD is an important clinical event that affects over 1500 people per million population in countries like Japan, Taiwan, and the US, with approximately two-thirds of them requiring hemodialysis, a quarter undergoes kidney transplantation, and only one-tenth go through peritoneal dialysis [9]. ESRD is the irreversible decline in kidney functions, which could be proved fatal without dialysis or transplantation [10].

Cardiovascular disease is a principal cause of mortality and morbidity in patients on hemodialysis, with up to 30–40% of all deaths attributed to it in the US renal data system [11]. Atherosclerosis is the primary reason behind cardiovascular disease, including heart failure, myocardial infarction, and stroke. When compared with healthy people, HD patients exhibited severe atherosclerosis in the carotid artery, as shown by measuring the carotid intima-media thickness (CIMT) using B-mode ultrasound. A measurement of over 0.9 mm is considered a marker for general atherosclerosis. It was previously shown that HD patients show increased CIMT, arterial stiffness, coronary artery calcification, and characteristic carotid plaques [11].

Atherosclerotic lesions occur early in renal dysfunction with peripheral arteries intense vascular wall thickening. The heightened cardiovascular disease risk in renal disease is probably the reason for high morbidity and mortality. The risk of death from CAD is in direct relationship to renal function deterioration in CKD patients that even a modest decrease in glomerular filtration rate in CKD stage two could lead to a significant increase in the risk of CAD. In HD patients, this increases from up to 100 times when compared with the general population [12, 13].

Melatonin receptors *MTNR1A* and *MTNR1B* SNPs have been linked to autoimmune diseases, including Graves' disease in a study on 83 Hashimoto's thyroiditis showing variations of rs2119882 of *MTNR1A* supporting melatonin pathway involvement in Graves' disease pathogenesis [14].

*MTNR1B* rs10830963 GG genotype is linked to increased fasting blood sugar levels, suggesting a significant relationship between melatonin, *MTNR1B,* and fasting blood sugar in type 2 diabetes [15].

In a recent study, evidence has been provided showing the relationship between rs2119882 and rs10830963 melatonin receptor polymorphism and lipid metabolism disorder in PCOS patients, highlighting the importance of the therapeutic and protective effects of melatonin and its metabolites [16].

The human *MTNR1A* gene, located on the chromosomal region 4q35.2, spans a genomic region of 21 913 bases and is split into two exons [17]. SNP rs13140012 in intron 1 of the *MTNR1A* gene can hinder the binding affinity of transcription factors and might help in predicting aggressive phenotypes of UCC [18]. It has also been related to a higher risk of oral cancer with synergistic effects of environmental factors [19].

Calcium nephrolithiasis was significantly associated with rs13140012. This common condition where calcium oxalate and calcium phosphate salts are predominant in the crystalline formation of kidney stones. The formation of these stones is dependent on an interplay between hormonal, genetic, and environmental factors as melatonin has been hypothesized to have a role in regulating tubular renal functions through the specific melatonin receptors in the renal cortex [17].

Aim: To study the relationship between melatonin levels and melatonin membrane receptor 1A (*MTNR1A*) SNP (rs13140012) in ESRD patients in Alexandria, Egypt, on maintenance hemodialysis with or without atherosclerosis.

## 2 Subjects and methods

### 2.1 Subjects

This case-control study was carried out on 80 subjects divided into 2 two groups: **Group I (patients group)**: It included 40 ESRD patients on regular hemodialysis. Their mean age was 47.25 ± 11.56 years, 22/40 (55%) were males. The patients' group was subdivided into two subgroups according to their CIMT into atherosclerotic and non-atherosclerotic subgroups. **Subgroup I A:** This Included 20 ESRD patients with atherosclerosis. **Subgroup I B**: This included 20 ESRD patients with no atherosclerosis. ESRD patients were enrolled from the Dialysis Unit at the Department of Internal Medicine, Nephrology Unit, Faculty of Medicine, Alexandria University, Egypt. Enrolment began in January 2019 and concluded in June 2019.

**Exclusion criteria:** Patients with Diabetes, Dyslipidemia, Malignancy, Active infection/inflammation, Patients on Beta-blockers, Patients using Melatonin supplements were excluded.

**Group II (Control group)**: It included 40 normal healthy volunteer subjects. Their mean age was 46.35 ± 9.48 years. They were matched for age and sex with group I.

### 2.2 Methods

#### 2.2.1 Sampling

Nine milliliters of whole blood was collected by venipuncture from an antecubital vein under complete aseptic technique into three vacutainer tubes:

a. Two mL in BD vacutainer® blood collection tubes containing K2EDTA for molecular analysis to detect *MTNR1A* gene SNP *(rs13140012).* Samples were transferred to the laboratory immediately.
b. Two mL in another BD vacutainer® blood collection tubes containing K2EDTA used for complete blood count (CBC).
c. Five mL in BD Vacutainer® plain blood collection tubes for melatonin assay, and chemical analysis. Blood was left to clot at room temperature for 15 min, followed by centrifugation at 4000 rpm for 10 min to separate serum. The serum used for melatonin analysis was stored at -80 °C for further use.

Samples were collected between 8 am, and 9 am. Patients' samples were withdrawn before their first dialysis session of the week. Neither patients or controls received treatments during sample collection.

#### 2.2.2 Routine laboratory investigations

All subjects included in the study were subjected to laboratory investigations including serum calcium., PTH, blood urea, serum creatinine, serum albumin, serum K, serum phosphorous, C reactive protein, total cholesterol, triglycerides, complete blood picture [20].

#### 2.2.3 Measurement of serum melatonin in (pg/mL)

Melatonin was analyzed in serum samples using the melatonin ELISA kit (IBL International, Germany) according to the manufacturer's protocol. First, (0.5 ml) of samples, Standards (0, 3, 10, 30, 100, and 300 pg/mL of melatonin), and Controls were passed through a C18 reversed-phase column and extracted with methanol. Evaporation was then done, followed by the reconstitution of the extract with water. Each extracted standard extracted control, and extracted sample (50 μL) was added to the respective wells of the Microtiter Plate, which was coated with anti-rabbit IgG (goat, polyclonal). Then, 50 *μ*L of melatonin biotin and 50 *μ*L of rabbit-antiserum were added into each well, shaken carefully, and incubated overnight (20 h) at 2–8 °C. After washing, 150 *μ*L of freshly prepared enzyme conjugate was added into each well and incubated for two hours at room temperature on an orbital shaker (500 rpm). The reaction was developed using 200μL p-nitrophenyl phosphate Substrate Solution. Optical densities were measured at 405 nm in an automatic microplate reader (Stat Fax 2100, Awareness Technologies).

Two quality controls were included in each run; Control 1 ranging from 5.7-14.9 pg/mL and Control 2 ranging from 58.2 - 99.1. According to the manufacturer's protocol, the intra-assay coefficients of variation for serum was 3.0–11.4% in the range of 8.8–151.7 pg/mL, while the inter-assay coefficients of variation were 6.4–19.3% in the range of 5.6–134.3 pg/mL. The lower detection limit of the assay was 1.6 pg/ml.

#### 2.2.4 Melatonin membrane receptor 1A (*MTNR1A*) SNP (*rs13140012*) genotyping

Genomic DNA was extracted from whole blood samples using GeneJET whole blood Genomic DNA Mini Kit (Thermo Fisher Scientific, USA) according to the manufacturer's instructions. DNA's purity and concentration were measured using Nanodrop 2000/2000c spectrophotometer (Thermoscientific, USA).

Genotyping for *MTNR1A* polymorphism (rs13140012) was performed using the 5'nuclease allelic discrimination assay. The PCR reaction mix included 10 μLTaqMan® Universal PCR Master Mix (Thermo Fisher Scientific, USA), 1 μL of TaqMan® SNP Genotyping Assay 20x (Assay ID: C__31861431_10), 20 ng DNA/reaction and DNAase free water to a final volume of 20 μL. Thermal cycling was done using Stratagene Mx3000P (Thermo Fisher Scientific, USA) as follows; 95 °C for 10 min for initial AmpliTaq Gold enzyme activation and 45 cycles of denaturation for 15 s at 95 °C and annealing/extension for 1 min at 60 °C. No template control (NTC) containing nuclease-free water was included in each run as a negative control. The fluorescence profile of each well was detected at the end of each cycle, and a graphic presentation of the fluorescence against the number of cycles was plotted. Data processing was performed using Stratagene Mx3000PTM Software (MX.PRO software).

#### 2.2.5 Carotid Doppler intima media thickness in mm [21]

CIMT is known as a low-level echo grey band which does not project into the artery lumen and was measured at the diastolic phase as the distance between the leading edge of the first and second echogenic lines of the far walls of the distal segment of the common carotid artery, the carotid bifurcation, and the internal carotid artery on both sides. The doppler was done with a duplex ultrasound system with a 7.5 MHz scanning frequency in the B-mode, pulsed Doppler mode, and color mode.

The B-mode scanning included the scanning of the left and right common carotid arteries (3 cm before the carotid bifurcation), carotid bifurcation, and the internal carotid artery 2 cm distally from the carotid bifurcation. CAIMT measurements were done in arterial segments without plaques. All examinations and measurements were completed by the same examiner to have examiner bias excluded.

#### 2.2.6 Statistical analysis of the data

Data were fed to the computer and analyzed using IBM SPSS software package version 20.0. (Armonk, NY: IBM Corp) Qualitative data were described using number and percent. The Kolmogorov-Smirnov test was used to verify the normality of distribution Quantitative data were described using range (minimum and maximum), mean, standard deviation, median, and interquartile range (IQR). Obtained results significance was judged at the 5% level.

The used tests were Chi-square test, Monte Carlo correction, Student t-test, Mann Whitney test, Kruskal Wallis test, Spearman coefficient, Odd ratio (OR), and Hardy-Weinberg.

### 2.3 Ethical approval

All procedures performed in studies involving human participants were per the ethical standards of the institutional and national research committee and with the 1964 Helsinki declaration and its later amendments or comparable ethical standards.

### 2.4 Informed consent

Informed consent was obtained from all individual participants included in the study.

## 3 Results

### 3.1 Statistics of the studied participants

No statistically significant differences were observed between ESRD patients and controls as regards age (P = 0.704) or gender (P = 0.116). ESRD patients included 22 (55%) males and 18 (45%) females, whereas the control group consisted of 15 (37.5%) males and 25 (62.5%) females. The age of ESRD patients had a mean of 47.25 ± 11.56, while in the control group, it was 46.35 ± 9.48.

ESRD had significantly higher parathormone hormone, urea, creatinine, potassium, phosphorous, c-reactive protein, triglycerides and total cholesterol than the control group. ESRD had significantly lower hemoglobin, and white blood cells than the control group. There was no significant difference between the two groups as regards albumin and platelets. Laboratory findings are summarized in Table 1.

**Table 1:** Demographic data and laboratory investigations of the studied groups.

	Patients (n = 40)	Control (n = 40)	p
Age (years)	47.25 ± 11.56	46.35 ± 9.48	0.704
Sex			
Male	22 (55%)	15 (37.5%)	0.116
Female	18 (45%)	25 (62.5%)	
Calcium (mg/dL)	8.73 ± 0.70	8.96 ± 0.32	0.070
Parathormone hormone (ng/L)	418.5 (8.81–3381.0)	32.0 (13.0–60.0)	<0.001∗
Urea (mg/dL)	157.7 ± 33.71	23.20 ± 7.06	<0.001∗
Creatinine (mg/dL)	10.26 ± 1.82	0.64 ± 0.14	<0.001∗
Albumin (g/dL)	4.33 ± 0.38	4.41 ± 0.43	0.411
Potassium (mEq/L)	5.96 ± 0.79	4.17 ± 0.41	<0.001∗
Phosphorous (mg/dL)	4.42 ± 1.61	3.21 ± 0.45	<0.001∗
C-reactive protein (mg/dL)	6.25 (1.20–125.5)	3.05 (0.50–6.40)	<0.001∗
Total cholesterol (mg/dl)	158.7 ± 34.04	133.7 ± 40.70	0.004∗
Triglycerides (mg/dl)	122.1 ± 20.66	99.48 ± 22.94	<0.001∗
Hemoglobin	10.36 ± 1.91	14.59 ± 1.19	<0.001∗
White blood cells	6.65 ± 2.0	7.72 ± 1.96	0.019∗
Platelets	220.8 ± 59.17	201.1 ± 28.32	0.063

Qualitative data were described using number and percentage and were compared using χ^2^or Fisher Exact test. Normally quantitative data were expressed using Mean ± SD and compared using the student t-test. While abnormally quantitative data were expressed using the Median (Min. – Max.) and were compared using the Mann Whitney test.

∗ Statistically significant at p ≤ 0.05.

### 3.2 Serum melatonin in the two studied groups

**Figure 1:**
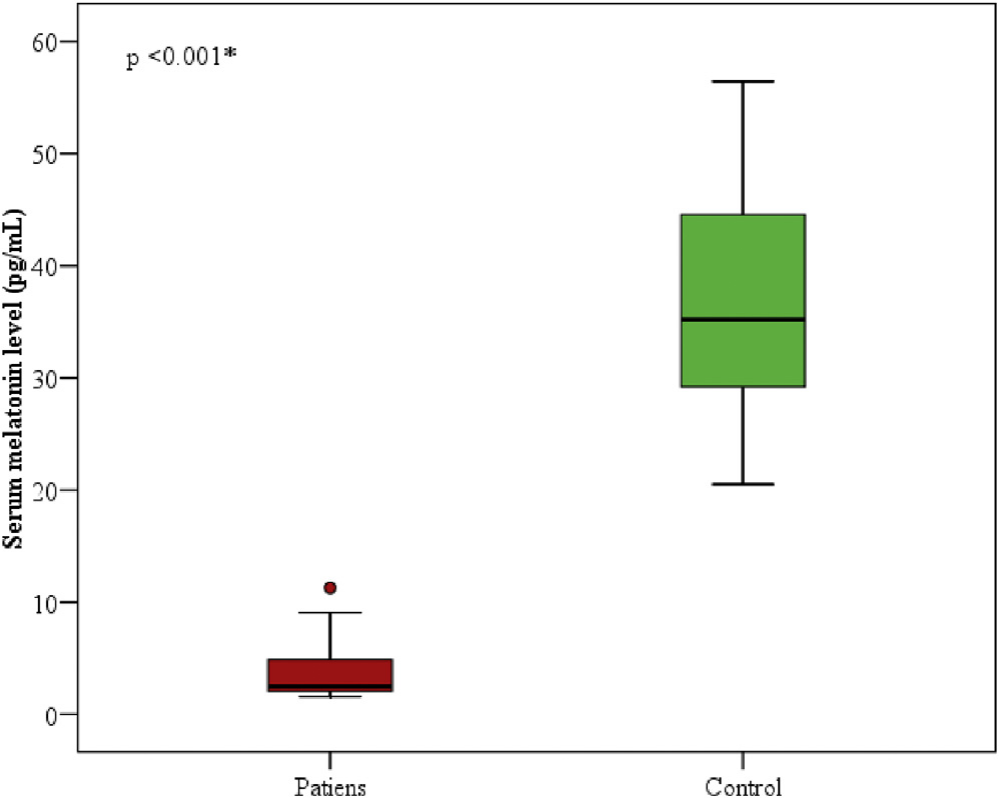
Comparison between the two studied groups according to serum melatonin (pg/mL).

Serum melatonin levels in the patients' group ranged from 1.6 to 11.30 (pg/mL) with a median of 2.5. While it ranged from 20.50 to 56.40 (pg/mL) in the control group with a median of 35.20. There was a statistically significant decrease in the patients' group in comparison to the control group (p < 0.001), Figure 1.

### 3.3 Melatonin membrane receptor 1A (*MTNR1A*) SNP (*rs13140012*) genotyping in the two studied groups

Table 2 showed the distribution of *MTNR1A* SNP (*rs13140012*) genotypes and alleles in the two studied groups. There was no statistically significant difference between the two groups for SNP rs13140012 and its alleles.

**Table 2:** Comparison between the two studied groups according to *MTNR1A* SNP (*rs13140012*) genotypes and alleles distribution.

*MTNR1A* SNP (*rs13140012*)	Patients (n = 40)		Control (n = 40)		p	OR (95%CI)
	No.	%	No.	%		
SNP						
AA®	2	5.0	1	2.5	-	1.00
AT	21	52.5	29	72.5	0.419	0.362 (0.031–4.260)
TT	17	42.5	10	25.0	0.889	0.850 (0.068–10.61)
Allele						
A®	25	31.25	31	38.75	-	1.00
T	55	68.75	49	61.25	0.321	1.39 (0.724–2.673)

OR: Odds ratio, R: Reference or wild type.

CI: Confidence interval, LL: Lower limit, UL: Upper Limit.

### 3.4 HWE

Table 3 demonstrated the patients group as consistent with Hardy-Weinberg equilibrium while the control group was not.

**Table 3:** A simple calculator to determine whether observed genotype frequencies of *MTNR1A* SNP (*rs13140012*) are consistent with Hardy-Weinberg.

*MTNR1A* SNP (*rs13140012*)	Observed	Expected	χ^2^	p
Patients (n = 40)				
AA®	2	3.9	1.968	0.161
AT	21	17.2		
TT	17	18.9		
Control (n = 40)				
AA®	1	6.0	11.122∗	<0.001∗
AT	29	19.0		
TT	10	15.0		

If P < 0.05 - not consistent with HWE.

Not accurate if < 5 individuals in any genotype group.

### 3.5 Serum melatonin and *MTNR1A* SNP (*rs13140012*) in the patients group

Table 4 demonstrated no significant association between (*MTNR1A*) SNP (*rs13140012*) with serum melatonin levels in the patients' group.

**Table 4:** Association between serum melatonin and (*MTNR1A*) SNP (*rs13140012*) in the patients' group.

*MTNR1A* SNP (*rs13140012*)	N	Serum melatonin		p
		Min. – Max.	Median	
SNP				
AA®	2	3.10–32.10	5.60	0.372
AT	21	1.90–56.40	27.10	
TT	17	1.60–47.50	5.10	
Allele				
A®	25	1.90–11.30	2.50	0.787
T	55	1.60–11.30	2.50	

Abnormally quantitative data were expressed using the median (Min. – Max.) and were compared using Mann Whitney or Kruskal Wallis test.

R: Reference or wild type.

p: p-value for the association between serum melatonin and rs13140012.

### 3.6 Carotid intima-media thickness (CIMT)

Table 5 demonstrated CIMT measurements in the patients and control groups. In the patients' group, CIMT ranged from 0.4 to 2.20 mm with a median of 0.90. In the control group, it ranged from 0.40 to 0.80mm, with a median value of 0.6. There was a statistically significant difference between both groups (p < 0.001).

**Table 5:** Comparison between the two studied groups according to CIMT.

	Patients (n = 40)	Control (n = 40)	p
CIMT	0.90 (0.40–2.20)	0.60 (0.40–0.80)	<0.001∗

Abnormally quantitative data were expressed using the median (Min. – Max.) and were compared using Mann Whitney.

p: p-value for the association between patients and control.

∗ Statistically significant at p ≤ 0.05.

### 3.7 CIMT and atherosclerosis in the patients' subgroups

CIMT in the non-atherosclerotic subgroup ranged from 0.4 – 0.8mm with a median value of 0.60. While in the atherosclerotic subgroup, it ranged from 1.0 – 2.2 with a median value of 1.0. There was a statistically significant difference between subgroups (p < 0.001).

### 3.8 Serum melatonin and atherosclerosis

Melatonin levels in non-atherosclerotic patients ranged from 2.0 – 11.30, with a median of 4.9. While in the atherosclerotic subgroup, it ranged from 1.6 – 2.50, with a median value of 2.30. There was a significant statistical difference (p < 0.001).

### 3.9 Atherosclerosis and SNP rs13140012 and its alleles in patient subgroups

Table 6 showed the genotype distribution of *MTNR1A* SNP (*rs13140012*) and its alleles frequency in the patients' subgroups according to whether patients were atherosclerotic or not. There was no statistically significant difference between the two subgroups for SNP rs13140012 and its alleles.

**Table 6:** Relation between atherosclerosis and *MTNR1A* SNP (*rs13140012)* in the patients' subgroups (atherosclerotic and non-atherosclerotic).

*MTNR1A* SNP (*rs13140012*)	Atherosclerosis				χ^2^	p
	No		Yes			
	No.	%	No.	%		
SNP	(n = 20)		(n = 20)			
AA®	2	10.0	0	0.0	1.770	^MC^p = 0.585
AT	10	50.0	11	55.0		
TT	8	40.0	9	45.0		
Allele	(n = 40)		(n = 40)			
A®	14	35.0	11	27.5	0.524	0.469
T	26	65.0	29	72.5		

χ^2^: Chi-square test MC: Monte Carlo, R: Reference or wild type.

p: p-value for the association between Atherosclerosis and rs13140012.

### 3.10 Serum melatonin and CIMT

**Figure 2:**
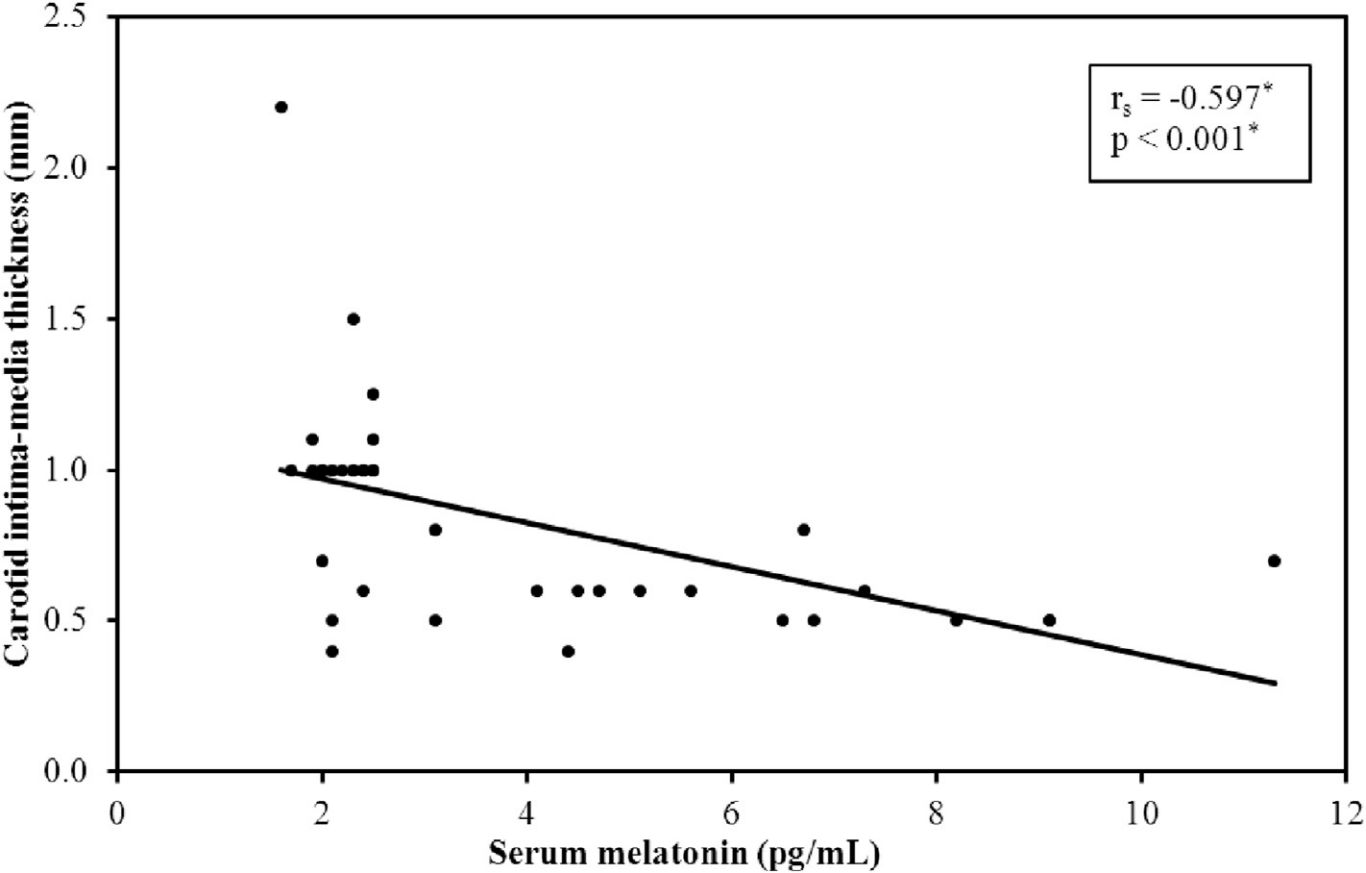
Correlation between serum melatonin and CIMT (n = 40).

There was a significant negative correlation between CIMT and serum melatonin levels (p-value <0.001), Figure 2.

### 3.11 CIMT and SNP rs13140012

There was no association between CIMT and *MTNR1A* SNP (*rs13140012*) as demonstrated in Table 7.

**Table 7:** Association between CIMT and *MTNR1A* SNP (*rs13140012*) in the patients' group.

*MTNR1A* SNP (*rs13140012*)	N	CIMT		p
		Range	Median	
SNP				
AA®	2	0.60–0.80	0.70	0.835
AT	21	0.40–1.10	1.0	
TT	17	0.40–2.20	1.0	
Allele				
A®	25	0.40–1.10	0.80	0.652
T	55	0.40–2.20	1.0	

Abnormally quantitative data were expressed using the median (Min. – Max.) and were compared using Mann Whitney or Kruskal Wallis test.

R: Reference or wild type.

p: p-value for the association between serum melatonin and rs13140012.

## 4 Discussion

Daytime melatonin levels in renal disease patients are contradictory as both increased and decreased melatonin levels have been noted in daytime hemodialysis patients [22, 23, 24, 25, 26].

In the present study, the serum melatonin levels in ESRD patients' group were statistically significantly decreased than their levels in the control group (P < 0.001).

The result of the present study was in agreement with Kock et al. [22] He and his colleagues suggested patients on daytime hemodialysis have shown that the nocturnal spike in melatonin above Dim Light Melatonin Onset (DLMO) is abolished in chronic renal failure. The melatonin rhythm was more likely to be diminished in hemodialysis patients than in patients with chronic renal insufficiency who were not, concluding that hemodialysis affects the rhythm [23]. Diminished melatonin levels are associated with more pronounced sleep disturbances in hemodialysis patients [27].

Karasek et al. also found decreased melatonin levels [28] in their study on two patients groups: The first group included compensated chronic renal failure patients, and the second group included ESRD patients. In both groups of patients with chronic renal disease, nocturnal melatonin levels were notably lower in comparison with healthy volunteers. It is also worth noting in patients with compensated renal failure; daytime melatonin concentrations were diminished.

Karasek et al. [28] stated it is still unclear how melatonin concentrations are affected in ESRD patients. Adrenergic function impairment that happens in CRF could be one cause of this [29]. The adrenergic system plays a crucial part in melatonin secretion [30, 31]. Abnormalities in the autonomic nervous system have been observed in long term hemodialysis patients [32]. Decreased responses and densities of β2-adrenoceptors have been reported in CRF patients [33]. In addition, uremic patients have significantly decreased β1-and β2-adrenoceptors [34]. It is also worth noting that in rats rendered uremic by partial nephrectomy. Serotonin N-acetyltrasferase, the primary enzyme in melatonin biosynthesis, showed decreased activity [30, 31, 35]. Low serum melatonin before and after hemodialysis and insufficient circadian melatonin profiles suggest the key reason for decreased melatonin production is probably caused by uremic toxins. Also, melatonin binds to alpha-1-acid glycoprotein and albumin, and so is not dialyzable when in that state [36]. Even though patients did not take β-blockers in the study one day before melatonin measurement, most of them took β-blockers before that to treat hypertension. The effects of long-term β-blockers usage could not be excluded [28].

Varizi et al. [24] measured serum melatonin in ESRD patients between 6 and 9 am. during hemodialysis and on an off-dialysis day. Pre-dialysis serum melatonin at 6 am. in the ESRD patients was comparable with the control group. Melatonin levels in the ESRD group dropped by about 25% during dialysis. Off-dialysis day changes during the same period were more or less the same as during dialysis.

Contrary to the result of the present study, Viljoen et al. [25] tested the melatonin status of CKD patients by measuring daytime plasma melatonin levels and by examining melatonin circadian rhythm. Plasma melatonin concentration was significantly increased in all CRF patient groups in the study, including those on conservative medical treatment and the ones on maintenance hemodialysis and those on peritoneal dialysis. Patients with successful transplantations had a significant decrease in melatonin levels. The circadian rhythm of melatonin secretion was limited in CRF with an absent nocturnal secretory surge in all HD patients and 80% of the post-transplantation patients.

Also, in Ludemann et al. [26] study with 35 ESRD patients, measuring the serum concentrations of melatonin, morning serum samples were taken from a control group with normal renal functions. Patients underwent dialysis for about 4 h between 7 am and 1 pm (S1 group) and between 1 pm and 8 pm (S2 group), or between 6:30 pm and 10:30 pm (S3 group). Mean melatonin concentration before hemodialysis was a lot higher than the control group (40.6 vs. 6.7 pg/mL; P < 0.001). Even though melatonin levels dropped to 20.3 pg/mL after dialysis, it was still higher than the control level. A diurnal rhythm for melatonin was identified in ESRD patients (P < 0.05), denoting that the patient's renal condition does not affect normal synthesis rhythm. The authors concluded that in ESRD patients, hemodialysis cannot normalize melatonin levels and that elevated concentrations of melatonin cause some secondary disorders in ESRD. Their theory behind the elevated melatonin levels was that physical principles of dialysis were based on small molecules exchanged via a semi-permeable membrane into a low osmolality fluid and that this would not apply to larger molecules besides melatonin binding to α1-acid glycoprotein and albumin and is not dialysable in that form [32].

To further support the hypothesis of elevated melatonin levels in ESRD patients, Ludemann et al. claim the disturbed hypothalamus-pituitary-gonadal axis disturbance often encountered in ESRD patients could be caused by elevated levels of melatonin linked to hypogonadism and amenorrhea [37, 38].

Several mechanisms could explain the circadian melatonin rhythm abnormalities in renal disease patients. As previously mentioned, daytime dialysis can lead to daytime sleepiness and nocturnal insomnia [29, 39]. This disturbed sleep-wake rhythm could lead to an absent trigger to start melatonin production at night [22].

Decreased melatonin levels in renal impairment patients have been linked to a derangement in β-adrenoreceptor-mediated responsiveness [23, 39]. The adrenergic system is associated with NAT synthesis [39, 40], an essential enzyme in melatonin biosynthesis. Nocturnal levels of NAT activity declined in partial nephrectomy [41].

The present study also found that the level of serum melatonin was significantly lower in ESRD patients with atherosclerosis compared to ESRD patients without atherosclerosis (p-value <0.001).

In a study by Bozkart et al. [42], 62 patients diagnosed with varying degrees of erectile dysfunction (ED) and 22 healthy individuals were included. The serum melatonin levels were performed. It was found that serum melatonin levels in ED patients were found to be much lower than controls. Sawada et al. [43] reported that after melatonin treatment (20 mg/kg per day for eight weeks), erectile responses were restored, with protecting contractile and relaxant responses, and there was increased neuronal and endothelial NO synthase (NOS) and decreased inducible NOS expression. Ischemic erectile tissue dysfunction has multiple mechanisms and risk factors, including hypoxia, chronic nutrient deficiency, and metabolic waste, and due to cytotoxicity, it could affect NO production or function [44]. Sawada et al. [43] also observed that the down-regulation of eNOS and nNOS expression might cause ED. eNOS and nNOS proteins were decreased in chronic ischemic CC tissue; those levels improved after melatonin treatment. Melatonin's protective effects on chronic ischemic CC by is by scavenging free radicals and via anti-oxidative properties [42].

Low levels of melatonin were noticed in type 2 diabetes patients [45] and hypertension [46]. It has also been demonstrated that blood melatonin levels are linked to cardiovascular disease severity [46]. These studies demonstrated that melatonin deficiency could exert its effects on different systems and is involved in various pathologies.

Javanmard et al. [47] reported that melatonin could have a positive influence on endothelial dysfunction even in severe cases of atherosclerosis. In that study, vascular cell adhesion molecule, C- reactive protein, and the mean levels of intercellular adhesion molecule decreased after four weeks of melatonin treatment. Higher serum NO levels in the study group were noted compared to the control group. It was hypothesized that melatonin might have a part in reducing endothelial cell damage markers and increasing vasodilator cytokines.

In another study by Jeong et al. [48] 12 healthy adult men whose vascular endothelial function [flow-mediated dilation (FMD)] was assessed, a comprehensive assessment of vascular health including brachial arterial blood pressure, FMD, and arterial stiffness (carotid-femoral pulse wave velocity; PWV) was assessed in 4 individuals at baseline and 1 h following a single oral dose of melatonin (5 mg). FMD (%) was positively associated with total overnight melatonin production and melatonin excretion rate. Compared with baseline, PWV significantly decreased (p = 0.003), and FMD increased (p = 0.173) 1 h after taking supplemental melatonin. Supplemental melatonin did not affect blood pressure. This new information shows that higher endogenous melatonin production is linked to better vascular endothelial function. Also, a single dose of supplemental melatonin can decrease arterial stiffness and improve endothelial function. This suggests that melatonin could be of benefit in regulating vascular function in humans. Achieving adequate overnight melatonin production may represent an important therapeutic strategy for the maintenance of optimal vascular health.

The present study did not find a significant association between SNP rs13140012 and atherosclerotic ESRD patients (p-value ranging from 0.067 to 0.564 among different alleles) despite the significant association between the same melatonin receptor SNP with recurrent and idiopathic nephrolithiasis in the study by Esposito et al. in 2011 [17]. Their study confirms that the *MTNR1A* gene is expressed in the kidney and supports the idea that the biological melatonin system may be involved in renal physiology and the pathogenesis of nephrolithiasis. It is worth noting a circadian variation in the urinary excretion of different solutes has been documented in healthy individuals: notably, the nocturnal excretion rates of potassium, sodium, chloride, and urate are only 50% of the rates observed during the light period [49], whereas phosphate reabsorption at night is more than that during the day [50]. Also, urinary pH and calcium showed a circadian variation in healthy subjects [51] In patients known to form stones; it has been demonstrated there is impaired circadian rhythmicity of urinary volume and some solutes excretion [52, 53, 54, 55, 56]. This was the first genetic polymorphism investigation to link the MTNR1A–biological melatonin system to calcium nephrolithiasis. The authors affirmed it would be essential to study these relationships before generalizing the findings in the study [17].

## 5 Conclusions

These results lead us to suggest that melatonin production is impaired in ESRD patients (included in this pilot study), and this impairment is even more evident in atherosclerotic ESRD patients.

## 6 Recommendations

Melatonin research on ESRD patients is an area that still requires further research to study its correlation with kidney disease and its complications.

Further studies on larger patient groups should be conducted on melatonin membrane receptor 1A (*MTNR1A*) SNP (rs13140012) in renal diseases.

## Declarations

### Author contribution statement

E. Elsayed: Conceived and designed the experiments; Performed the experiments; Analyzed and interpreted the data; Contributed reagents, materials, analysis tools or data.

T. Salem: Conceived and designed the experiments; Analyzed and interpreted the data; Contributed reagents, materials, analysis tools or data.

M. El Nashar: Performed the experiments; Wrote the paper.

N. El Kholy and A. El Aghoury: Analyzed and interpreted the data; Contributed reagents, materials, analysis tools or data.

### Funding statement

This work was supported by 10.13039/501100002353Alexandria Faculty of Medicine, Alexandria University.

### Competing interest statement

The authors declare no conflict of interest.

### Additional information

No additional information is available for this paper.
